# (Cost-)effectiveness of an internet-based physical activity support program (with and without physiotherapy counselling) on physical activity levels of breast and prostate cancer survivors: design of the PABLO trial

**DOI:** 10.1186/s12885-018-4927-z

**Published:** 2018-11-06

**Authors:** H. J. van de Wiel, M. M. Stuiver, A. M. May, S. van Grinsven, N. K. Aaronson, V. P. Retèl, H. S. A. Oldenburg, H. G. van der Poel, S. Horenblas, W. H. van Harten, W. G. Groen

**Affiliations:** 1 Division of Psychosocial Research and Epidemiology, The Netherlands Cancer Institute, Amsterdam, The Netherlands; 2 Center for Quality of Life, The Netherlands Cancer Institute, Amsterdam, The Netherlands; 3 ACHIEVE Centre of Applied Research, Faculty of Health, University of Applied Sciences Amsterdam , Amsterdam, The Netherlands; 4 Julius Center for Health Sciences and Primary Care, University Medical Center Utrecht, Utrecht of University, Utrecht, The Netherlands; 5 Rijnstate Hospital, Arnhem, The Netherlands; 6 Department of Health Technology and Services Research, University of Twente, Enschede, The Netherlands; 7 Division of Surgical Oncology, The Netherlands Cancer Institute, Amsterdam, The Netherlands; 8 Department of Urology, The Netherlands Cancer Institute, Amsterdam, The Netherlands

**Keywords:** Physical activity, Internet-based intervention, Breast cancer survivors, Prostate cancer survivors, RCT

## Abstract

**Background:**

Higher levels of physical activity (PA) after treatment are associated with beneficial effects on physical and psychosocial functioning of cancer survivors. However, survivors often do not meet the recommended levels of PA. In order to promote PA, we developed a closed internet-based program. The aim of the study is to evaluate the (cost-)effectiveness of an internet-based PA-promotion program, alone or combined with physiotherapy counselling, compared to usual care, on PA-levels of breast or prostate cancer survivors. In this multicenter randomised controlled trial (RCT), breast or prostate cancer survivors who completed their primary treatment 3–12 months earlier, will be randomised to either 6-months access to a fully-automated internet-based intervention alone, an internet-based intervention plus remote support by a physiotherapist, or a control group. The intervention is based on the Transtheoretical Model and includes personalized feedback, information, video’s and assignments. Additionally, in a second arm, physiotherapy counselling is provided through monthly scheduled and on-demand telephone calls. The control group will receive usual care and a leaflet with PA guidelines.

**Methods:**

At baseline, 6 and 12 months, the primary outcome (PA) will be measured during 7 consecutive days by accelerometers. Secondary outcomes are self-reported PA, fatigue, mood, health-related quality of life, and costs. The group differences for primary and secondary outcomes will be analyzed using linear mixed models.

**Discussion:**

If proven to be (cost)effective, this internet-based intervention, either alone or in combination with telephone support, will be a welcome addition to previous RCT’s.

**Trial registration:**

Netherlands trial register (NTR6911), Date of trial registration: December 21, 2017.

**Electronic supplementary material:**

The online version of this article (10.1186/s12885-018-4927-z) contains supplementary material, which is available to authorized users.

## Background

Due to improved diagnostics and treatment of cancer, the number of survivors is increasing. In 2008, 28.8 million people worldwide had survived cancer at least 5 years [1]. Breast and prostate cancer have the highest prevalence of all cancers among women and men, respectively, with an incidence of 364.400 and 359.900 in the European Union in 2012 [2]. After treatment, many survivors experience negative consequences such as pain, impaired physical functioning and fatigue [3].

Physical activity (PA) interventions have clear beneficial effects on physical and psychosocial functioning, both during and after cancer treatment [4–7] Furthermore, a systematic review of 100 studies showed that higher levels of PA are associated with a substantially lower risk of recurrence, and of overall and cancer specific mortality [8]. Risk reductions of 20–60% have been reported in breast and prostate cancer depending on the amount and intensity of PA [9, 10]. However, survivors often do not meet the recommended PA level of ≥150 min moderate to vigorous physical activity (MVPA) per week [11]. Low levels of PA often remain low for years after treatment [12, 13]. Since cancer survivors who adhere the PA recommendation save, on average, $4686.1 (1–5 years’ survival time) in health care costs [14], efforts need to be made to encourage PA in an effective manner, in order to improve health status among survivors.

In 2015, we developed an Internet-based Physical Activity Support program (IPAS) to improve physical activity [15] and tested it in lung [16] and breast [17] cancer survivors. In breast cancer, there were some indications for the impact of the program, but the study had limited power to detect differences in subgroups (eg. during and after completion of treatment) and the study lacked a control group.

In lung cancer patients the use was limited, and effects were minimal, mainly due to the intensive treatment [16]. To date, few well-powered studies have been done using a similar distance based approach and the efficacy is limited [18].

The IPAS intervention is based on theoretical frameworks that are associated with PA motivation and behaviour in cancer survivors [19]. A meta-analysis of 85 web-based studies showed that interventions with a strong theoretical base are more likely to be effective than interventions without or with a weak theoretical base [20]. Additionally, the results of the review indicated that adding extra communication options with a counsellor, either via telephone, text message or e-mail, adds to the effectiveness of a web-based intervention. In the IPAS feasibility study, no additional communication mode was included. In focus groups conducted during the feasibility study, many participants indicated that an offline counselling moments would be helpful [21]. Therefore, we added physiotherapist counselling by phone as an extra feature, which may further enhance the outcome without large increments in costs. The aim of the present study is to investigate the (cost-)effectiveness of IPAS on short- and long-term PA levels, and to explore the added value of monthly in-person support, compared to usual care.

**Table 1:** Concepts from TTM, TPB and specific SCT to target per stage

Stage	Targeted concepts per information set	Transtheoretical model	Theory of Planned Behaviour	Social Cognitive Theory
Stage 1 & 2	Knowledge & attitudes	Consciousness raising Dramatic relief Environmental reevaluation Self-reevaluation	Attitudes	Knowledge Outcome expectations
	Self-efficacy	Self-efficacy	Perceived behaviour control	Self-efficacy
Stage 3	Knowledge & attitudes	Consciousness raising Dramatic relief Environmental reevaluation Self-reevaluation	Attitudes	Knowledge Outcome expectations
	Self-efficacy	Self-efficacy	Perceived behaviour control	Self-efficacy
	Goals & rewards	Self-liberation Reinforcement management	–	Health goals
Stage 4 & 5	Goals & rewards	Self-liberation Reinforcement management	–	Health goals
	Social support & environment	Helpingrelationships Counterconditioning Stimulus control	Subjective norms	Social and physical environment

Legend: stage 1 = precontemplation; stage 2 = contemplation; stage 3 = preparation; stage 4 = action; stage 5 = maintainance

## Methods/Design

### Trial design

The PABLO study is a three-armed multicenter randomised controlled trial (RCT). The participants will be recruited from a specialized cancer hospital: the Netherlands Cancer Institute (NKI), Amsterdam, and a large teaching hospital: Rijnstate, Arnhem.

**Fig. 1:**
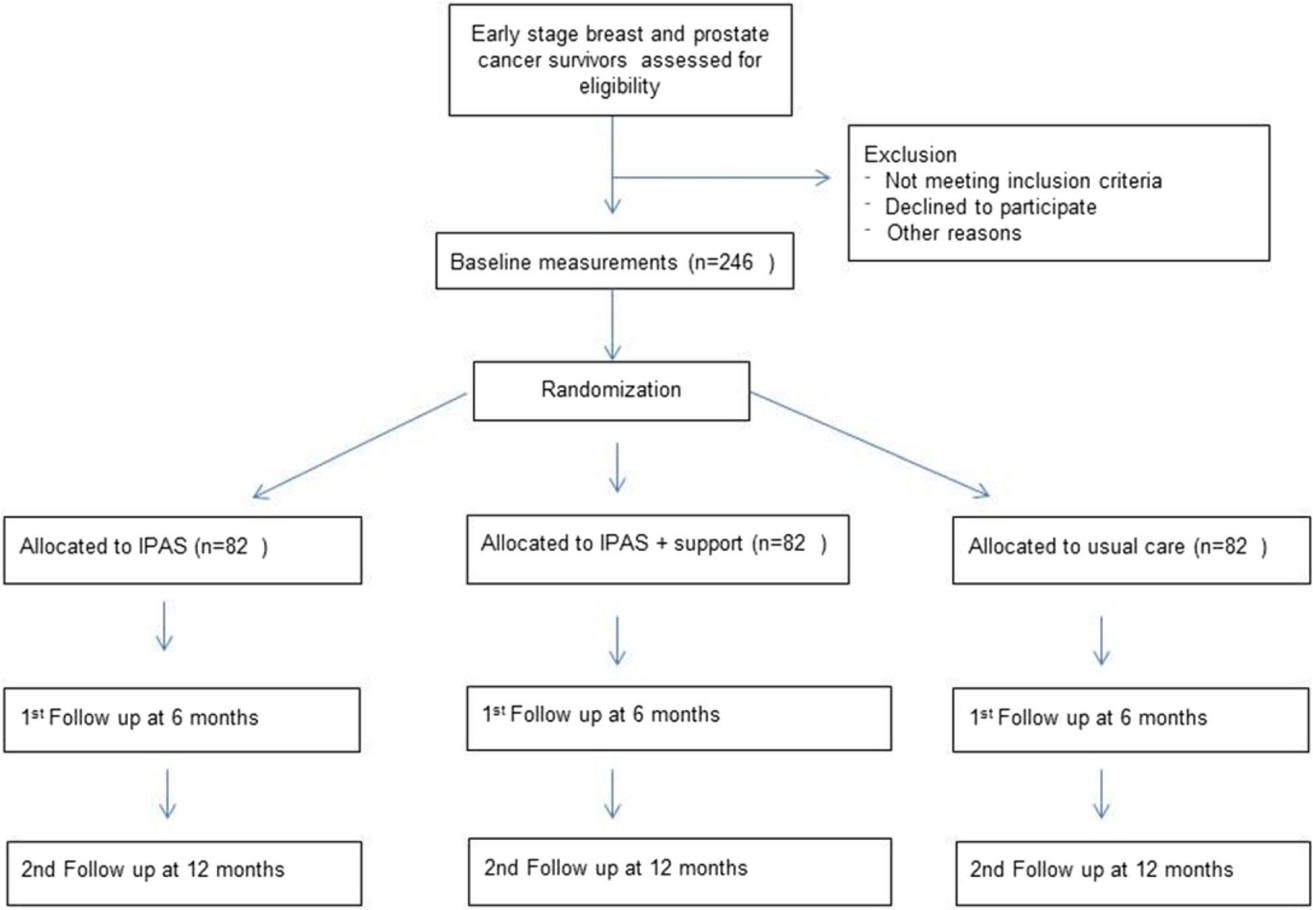
Flowchart of the PABLO-tudy

Participants will be randomised to one of the three study arms. Participants allocated to the first intervention arm will receive 6-month access to the intervention (IPAS). The second intervention arm will receive 6-month access to IPAS, as well as additional support by a monthly phone call with a physiotherapist (IPAS+TS). The control group will receive usual care and a leaflet with PA guidelines. The total study duration per participant is 12 months. This protocol will follow the SPIRIT guidelines. Figure 1 contains the flowchart for the study.

### Aim and hypotheses

The primary aim of the study is to evaluate the effectiveness of the IPAS program (with or without additional telephone support), compared to usual care, on objectively measured PA in patients with primary breast and prostate cancer who have completed primary treatment 3–12 months ago.

We have the following secondary aims: first, to evaluate the effect of the two interventions on self-reported PA, stage of change (regarding PA behaviour), fatigue, mood and health related quality of life compared to usual care. Second, to perform a cost-effectiveness analysis to estimate the expected allocative efficiency of IPAS and IPAS+TS vs. usual care. Third, to identify potential moderators (clinical and socio-demographic characteristics) and mediators (attitudes towards PA, social support and self-efficacy) of the intervention effect on PA levels. Finally, as an exploratory aim (as the study is not powered for this comparison), to evaluate the comparative effect of IPAS alone with IPAS+TS on objectively measured PA.

We hypothesize that using IPAS or IPAS+TS will increase levels of PA more than usual care where patients only receive a leaflet with current PA guidelines. Additionally, several studies have shown the value of personal feedback to increase cancer survivors’ PA levels [20–22]. Therefore, we hypothesize that participants in the IPAS+TS group will increase their PA-levels to a greater extent than participants receiving IPAS only. Additionally, we hypothesize that increased PA resulting from the exposure to IPAS or IPAS+TS will lead to decreased levels of fatigue, and improved HRQOL and mood as compared to control. Finally, we hypothesize that total costs for IPAS will be lowest compared to IPAS+TS, due to the low intervention costs, or somewhat higher compared to UC, and “gains” for IPAS−/+TS in terms of improved QoL.

### Inclusion and exclusion criteria

#### Inclusion criteria

Patients with histologically confirmed primary breast or prostate cancer (stages: T1 – T4, N0 – N3 and M0), who may currently be receiving adjuvant endocrine treatment (breast and prostate cancer) or Trastuzumab (breast cancer) will be included in the study. Primary treatment should have been completed a minimum of 3 months and a maximum of 12 months prior to study entry. All patients need to provide written informed consent.

#### Exclusion criteria

Main exclusion criteria are a lack of basic proficiency in Dutch, serious cognitive or psychiatric problems that would preclude following the intervention or completing the study questionnaires, and lack of internet access. Patients without a digital ID (DigID), which is a Dutch digital authentication code based on one’s social security number (mostly used for governmental services) will be excluded. DigID is one of the most frequently used authentication methods in the Netherlands. Also, patients participating in concurrent studies or rehabilitation programs containing psychosocial and/or exercise interventions will be excluded, as well as patients who are unable to perform unsupervised exercise at the recommended levels or who cannot safely perform such exercise according to the pre-exercise screening recommendations of the American College of Sports Medicine [23]. Patients who have (signs of) cardiovascular, metabolic, or renal disease, can only participate after approval by the treating physician. Lastly, because our aim is to increase PA-levels of cancer survivors, we will a-priori determine, by a short interview, who already meets the PA guideline of > 150 min MVPA/week of moderate to vigorous PA for more than 6 months. To take into account a ~ 30% overestimation of self-reported PA [24], we will be excluding patients who report > 200 min/week MVPA for longer than 6 months.

### Recruitment and eligibility

In total, 246 patients will be recruited. We will include patients both retrospectively and prospectively. For the prospective part, the treating medical specialist or specialized nurse practitioners will inform the patient about the study during a follow-up appointment in the hospital. The clinician checks eligibility with the aid of a screening-list of inclusion and exclusion criteria. Interested patients will receive an information package. After two weeks, the researcher will contact the patient by phone to confirm whether or not the patient wants to participate. For the retrospective recruitment, eligible patients will be identified from medical records by the treating physician and will be sent study information by mail. If approved via a response card, the researcher will contact the patient by phone to confirm participation or non-participation.

#### Non-participation analysis

Many trials on PA behaviour change interventions show recruitment rates of around 30% [25]. We expect that, in the current trial, a substantial percentage of eligible patients will also decline the invitation to participate. To gain more insight into the characteristics of this group, the non-participants will be invited to complete a one-time online questionnaire similar to the baseline questionnaire completed by study participants. In addition, we will ask them if they are willing to report their reason(s) for not participating.

### Randomisation

Patients will be allocated to one of the three study arms, using a minimization procedure that ensures balance of the groups in terms of tumor type (breast/prostate), hospital (NKI/Rijnstate), age (> 50, 50–60, > 60), and current endocrine treatment (yes/no). Participants will be randomised by the researcher, using a computer generated random assignment procedure (ALEA, [26]) to one of the two intervention groups (IPAS or IPAS+TS) or to the usual care control group with a 1:1:1 distribution. Neither participants nor researchers will be blinded to the randomization results.

### Interventions

#### IPAS

The Internet-based Physical Activity Support intervention is structured according to the Transtheoretical model (TTM) [27] and uses aspects from the Theory of Planned behaviour [28] and Social Cognitive theory [29] (Table 1). The TTM postulates that subjects can be classified into one of five stages of behavioural change related to the desired behaviour, in this case, meeting physical activity guidelines [27]. The five stages identified are [1] precontemplation (not sufficiently active and not intending to change), [2] contemplation (not sufficiently active but willing to change within next 6 months), [3] preparation (not sufficiently active but planning to change within 1 month), action (sufficiently active but for less than 6 months) or maintenance (sufficiently active for longer than 6 months). Patients can move through the stages during the intervention. In every stage, patients receive information that matches the particular stage. This approach is essentially the same as in the pilot intervention described previously [17], but has been improved in several ways to make it more attractive by adding more and more appealing information, images, interactive assignments and video’s.

During the six months of the intervention, patients will be invited by email to fill out an online questionnaire a month after completing the previous questionnaire. Questions will be posed on current PA levels (last 7 days) and motivation to become more physically active (see Additional file 1). Based on their responses, patients will be classified into one of the TTM stages, using the behaviour of ≥150 min per week of moderate to vigorous PA as target behaviour [30]. If patients forget to complete the questionnaire, a reminder will be sent after one week. After patients have completed their questionnaire, they will be directed to a content page that provides feedback on current PA levels via a graph and a table related to cancer survivorship guidelines (i.e. ≥150 min moderate to vigorous physical activity per week and two days a week of muscle strengthening exercises) [30]. Additionally, information is provided on several topics (e.g. “benefits of exercise”, “fatigue”, “start exercising”) in writing and through videos. The videos show breast or prostate survivors (depending on the participant’s diagnosis), who explain perceived benefits from exercise, or how they have overcome barriers to exercise. Other videos show physiotherapists or a physician explaining exercise principles and benefits. Also, there are several homework assignments (e.g., setting realistic goals, seeking social support, and identifying barriers and facilitators for being (more) physically active). Although not included in the criterion for target behaviour of ≥150 min of MVPA per week, throughout the program we also encourage patients to do muscle strengthening exercises at least 2 days a week, in accordance with current guidelines for physical activity for cancer survivors [31]. Table 1 presents an overview of the approach per stage of change, and examples of content are provided in Tables 2 and 3. Additional file 2 shows an example of the advice page that will be provided to the patients. New material will be provided monthly, tailored to the patients’ current stage of change. We have developed three content sets for every stage except for the maintenance stage for which we developed two content sets. Most of the content is the same for breast and prostate cancer survivors, but texts and videos are tailored to the specific population.

**Table 2:** Example of the provided content for the precontemplation stage

Stage 1 (precontemplation), content set 1			
Component	Targeted concept(s) (and processes of change)	Stage-appropriate strategy	Application
Information: Physical activity after cancer	Knowledge & attitudes (consciousness raising and dramatic relief)	Providing information on consequences of physical activity.	Introduction text with picture. Personal feedback on PA level of last week showed in graph and table. Written stories/quotes, Two movies (advice of a doctor and a personal testimonial of a survivor).
Assignment 1: Pros of physical activity	Knowledge & attitudes (consciousness raising)	Weighing pros and cons, emphasize pros.	Assignment on advantages of PA, including ticking options.
Assignment 2: Physical activity and significant others	Knowledge & attitudes (environmental reevaluation)	Environmental reevaluation (consequences to others).	Assignment on influence on others, including two text boxes.
Assignment 3: Past success	Self-efficacy	Prompt focus on past success (regarding exercise), with solution-focused questions.	Tips/examples, assignment on successes including text boxes.

**Table 3:** Example of the provided content for the action stage

Stage 4 (action), content set 1			
Component	Targeted concept(s) (and processes of change)	Stage appropriate strategy	Application
Information: Physical activity after cancer	Knowledge & attitudes	Providing information on consequences of physical activity via a link, providing information on how to (start) be(ing) physically active via a link and providing information on how to stay physically active.	Introduction text with picture. Personal feedback on PA level of last week showed in graph and table. Written stories/quotes, Two movies (advice of a physiotherapist and a personal testimonial of a survivor).
Assignment 1: Keep setting goals	Goals & rewards (self-liberation, reinforcement management)	Prompt trying out new activities, keep setting a goals and graded tasks, action planning and rewarding.	Information, Tips/examples, assignment on goals stetting, including text boxes.
Assignment 2: A bad week	Self-efficacy	Relapse prevention.	Information, Tips/examples, assignment on barriers and facilitators, including text boxes.
Assignment 3: Pros of being physically active	Self-efficacy	Prompt focus on past success including gained benefits, solution-focused questions.	Testimonial of a survivor, tips/examples, assignment on successes, including text boxes.

Apart from some obvious differences, e.g. showing a female patient as a role model for breast cancer survivors and a male role model prostate cancer survivors in the videos, we tailored information texts on the benefits of exercise to the available evidence for that group. Also, information on exercising with (risk of) lymphedema is only provided to breast cancer patients.

In total, the intervention lasts for 6 months. To facilitate and stimulate use of the IPAS, the intervention is embedded in the hospitals’ patient portal. Via this portal, patients can, for example, also access their medical record and see their clinical appointments. Patients receive two positively worded reminders via email in the first two months to stimulate their participation.

#### IPAS with telephone support (IPAS+TS)

In the second intervention group, the IPAS intervention will be complemented with additional monthly support provided by a physiotherapist (IPAS+TS). With the exception of the first appointment, which will be a face to face intake, consultations will be done by phone.

During the 45-min intake, a physiotherapist will briefly introduce the study and the IPAS intervention, and discuss current PA level and motivation and barriers to PA. Subsequently, the patient will be asked to exercise on a stationary bike or treadmill, to experience the desired moderate intensity. The workload on the bike/treadmill will be determined by the physiotherapist based on heart rate and clinical signs (breathing frequency, etc.) and confirmed by ratings of perceived exertion (RPE 11–13 on a 6 to 20-point Borg scale) [32]. The intake session is concluded by making an agreement with the patient on the intended behaviour change, and by establishing the most convenient time at which the patient can be reached by telephone. Telephone calls will be scheduled as soon as patients have filled out the monthly questionnaire in IPAS, and additional calls can be made on an ad-hoc basis, when desired. Prior to the telephone call, the physiotherapist will look-up the current stage of change by reviewing the last completed online questionnaire. During the monthly call, the physiotherapist first confirms the stage of change by discussing current PA levels. Next, the physiotherapist provides feedback on the stage of change and restates the agreed behaviour from the previous (intake- or telephone-) consultation. The patient is asked to comment on her or his experiences with respect to exercise behaviour in the past period. The physiotherapist will help the patient reflect on these experiences and accompanying thoughts, and will normalize, reinforce or explain physical activity behavior whenever relevant. The telephone consultation concludes with an agreement on a behavioural goal (related to physical activity) for the next month. Physiotherapists take notes of each discussion in standardized forms (see Additional file 3).

All physiotherapists receive a two-hour-training and detailed guidelines for the intake and telephone calls. Physiotherapists are instructed to choose their words carefully so as to avoid coming across as being judgmental or directive. Also, they are instructed to support patients in finding their own solutions to experienced barriers, to normalize negative experiences and to reinforce positive experiences, whenever possible.

#### Usual care

The control group will receive usual care and a leaflet with current physical activity guidelines for cancer survivors [31]. Both intervention groups will have access to the same leaflet via IPAS. The leaflet explains the physical activity guidelines and provides information on how the intensity of moderate to vigorous PA can be monitored by Borg rating of perceived exertion, a “talk-test” (when it becomes more difficult to talk in whole sentences) and heartrate self-monitoring. Although they are not actively referred, participants are not prohibited from participating in a rehabilitation or exercise programs with a physiotherapist in primary care, if they so wish. At 6 months, questions about participation in such programs will be included in the questionnaire.

### Sample size calculation

With a total sample of 195 patients (65 per group), the study will have 80% power to detect an effect size of 0.5 for the primary outcome (change in weekly time spent in MVPA) with a *p* value of 0.05. An effect size of 0.5 typically represents a group difference or change over time that is perceived as being clinically relevant [33].

In total, 246 subjects will be recruited for this study, to allow for an attrition rate of approximately 20% (i.e., subjects who discontinue participation in the study entirely, including failure to complete all follow-up measures; those who discontinue participation in one of the groups but complete the follow-up assessments will be included in the analysis). We did not chose for a forced 50–50% inclusion of breast and prostate cancer survivors. Eligible patients will be included, regardless of the tumor type. Thus, at least 195 subjects will be available for the primary intention-to-treat analysis.

**Table 4:** Outcome measures and measurement time points

Outcome	Measured by	Baseline	6 months	12 months	Continuously
PRIMARY					
Physical Activity	Objectively (Accelerometers and GPS-trackers)	x	x	x	
SECONDARY					
Physical Activity	Self-reported (IPAQ)	x	x	x	
Stage of behaviour change	Stage of change	x	x	x	
Fatigue	MFI	x	x	x	
Mood	POMS				
Quality of life	SF-36 and EQ5D	x	x	x	
OTHER OUTCOMES					
Cost outcomes	iMCQ iPCQ		x	x	
Social-demographic and clinical data	Social-demographic questions and patient medical file	x			
Moderators and Mediators of PA	Social-demographic questions and patient medical file	x			
Non-participation data	Reasons of non-participation question + Baseline questionnaire.	x			
Compliance	Questionnaire + Portal log data		x		x
Patient evaluation of the program	Questionnaire		x		
Contamination question for control group	Question on alternative PA program participation		x	x	

### Outcomes measures

#### Primary outcome

The main study parameter is objectively measured PA, using the Actigraph GT3X+ activity monitor (Actigraph, Pensacola, Florida, USA) and a BT-Q1000XT GPS-device (QStarz International Co). The Actigraph is a small tri-axial accelerometer that can measure accelerations from 0.05 to 2.00 G [34]. An instruction leaflet will be provided to explain how both devices should be worn on the right hip. The accelerometers and GPS devices will be initialized to collect data at 5 s intervals during 7 consecutive days. After each period of data collection (at baseline, 6 and 12 months) the devices will be sent back by patients to the research institute via mail. Output (activity counts and GPS data) will be downloaded to a PC after the monitoring period. Weekly time spent in MVPA will be calculated by standard cut-points [35, 36]. GPS-data will be used to better classify PA behaviour, e.g. if the speed of GPS data- points is < 12 km/h, the modality will be set to ‘walking & jogging’, and if the speed of the GPS data-points is ≥12 and < 25 km/h, modality will be set to ‘cycling’. Change in weekly time spent in MVPA between baseline and 6 and 12 months will be used as the main outcome parameter. An overview of primary and secondary outcome measures is provided in Table 4.

#### Secondary outcomes

Secondary outcomes are measured by questionnaires at baseline, 6 and 12 months follow-up. The cost questionnaires will be filled in after 6 and 12 months.

##### Self-reported PA level

The International PA Questionnaire (IPAQ) will be used to assess self-reported PA during the past seven days in five activity domains (i.e. job related, transportation, household activities, recreational activities and sedentary activities).

Total MET (Metabolic Equivalent of Task) score in MET*min-per-week (continuous score from the IPAQ scoring protocol) will be calculated following MET values per activity as proposed by Ainsworth et al. [37–39]. The IPAQ has been used previously in studies with cancer patients [40, 41], and has acceptable psychometric properties in terms of reliability and validity [42].

##### Stage of change

Stage of change is defined as motivational readiness towards a goal behaviour [27]. Participants will be asked: Which of the following statements fits you? 1) I’m not physicaly active and I’m not planning to become physicaly active within six months, 2) I’m not physicaly active and I’m planning to become physicaly active within six months, 3) I’m physicaly active but not regularly, 4) I’m physicaly active on a regular base but became this within the last six months, 5) I’m physicaly active on a regular base for more than six months [27].

##### Fatigue

Fatigue will be assessed with the Multidimensional Fatigue Inventory (MFI) [43]. The MFI consists of 20 items, categorized into five scales: general fatigue, physical fatigue, reduced activity, reduced motivation, and mental fatigue. The MFI is a valid and reliable instrument with internal consistencies ranging from 0.79 to 0.93 in cancer patients [44], which has been used in many studies with cancer patients as well as other populations [43].

##### Mood

Mood will be measured with the Profile Of Mood States (POMS) [45]. Mood will be measured with the shortened, 30-item version of the Profile of Mood States. Items are combined to form six separate subscales: tension-anxiety, depression-dejection, anger-hostility, vigour-activity, fatigue-inertia, and confusion-bewilderment. The subscale scores can be combined to form an overall measure of affect that is called total mood disturbance.

##### Health-related quality of life

We will measure HRQOL with the Medical Outcomes Study Short Form-36 (SF-36) [46]. This is the most widely used measure of general health status in clinical studies throughout the world. It generates scores in eight dimensions of HRQOL and two summary scores for physical and mental health. Additionally, for the purpose of CEA, we will administer the EQ5D-5 L. The EQ-5D health questionnaire is applicable to a wide range of health conditions and treatments. The instrument provides a simple descriptive profile and a single index value for health status. This health status is linked to utilities and is therefore used to calculate Qualify Adjusted Life Years (QALYs) for the cost-effectiveness analysis (CEA). The EQ-5D-5 L is primarily designed for self-completion by respondents and is ideally suited for use in postal surveys, in clinics and face-to-face interviews. It is cognitively simple, taking only a few minutes to complete [47].

##### Medical consumption questionnaire (iMCQ)

The iMCQ is a generic instrument for measuring medical costs. The iMCQ includes questions related to frequently occurring contacts with health care providers, which enables the estimation of the costs for the CEA from a societal perspective [48].

##### Productivity cost questionnaire (iPCQ)

The impact of disease on the ability of a person to perform work should be part of a cost-effectiveness analysis when a societal perspective is applied. The iPCQ is a generic instrument for measuring productivity (loss). The iPCQ includes questions related to the ability to perform and/or return to work after cancer diagnosis and treatment. In the current project, these question will only apply to patients who had a paid job at diagnosis [49].

#### Other outcomes

##### Socio-demographic and clinical data

The patients’ age, education, marital status, living situation, employment status, weight and height, medication and smoking status, and history of PA will be obtained via a questionnaire. Clinical information, including date of diagnosis, tumour characteristics, and treatment history (type of surgery, possible breast reconstruction, radiotherapy, chemotherapy, endocrine treatments) will be abstracted from the patients’ medical records. During the follow-up period, data on disease status (progression/recurrence), and any additional treatment will be obtained from the medical records.

##### Moderators and mediators of PA participation

Clinical and socio-demographic characteristics will be considered as moderators. As potentially mediating factors, data on behavioural variables related to PA will be collected through a questionnaire at baseline, 6 and 12 months. A series of questions will be asked regarding the attitudes towards PA, social support and self-efficacy. Patients’ preferences for type of exercise intervention will be assessed.

##### Non-participation analysis

We will ask non-participants if they would be willing to complete an online survey. If they consent, they will receive the survey containing the above questionnaires on socio-demographic and clinical characteristics, minutes of PA (IPAQ), stage of change, fatigue (MFI), mood (POMS), HRQOL (SF-36) and mediators of PA participation. We will also inquire about reasons for not participating. We will compare non-participants with trial participants, using baseline questionnaire data. This information can also be used for the cost-effectiveness analysis, to estimate the possible adherence and the real world use of the programs.

##### Compliance

For both intervention groups, the number of questionnaires completed during IPAS or IPAS+TS, the duration of log-ins and navigation behaviour in the PA program (which pages were visited and for how long) will be collected from log data, which is a standard feature of the software of the hospital information system. In IPAS+TS, the number, duration and discussed topics of the telephone counselling sessions will be registered in the protocol by the physiotherapist. Furthermore, patients will be asked to complete a short questionnaire related to self-reported use of IPAS at 6 months. Scoring options will be either constructed as a 5-point Likert scale or as open ended responses. All patients, including those in the control group, will be asked if they have pursued any (other) activities relating to increasing their levels of PA (e.g., use of internet resources, self-help text books, physical therapy, etc.), during the period of the study. We will also ask the participants via which medium they most visited IPAS: computer, tablet or mobile phone.

##### Patients’ evaluation of the intervention program

At the 6-month assessment, the patients in the two intervention groups will be asked about their experience with the internet-based PA program. They will be asked to complete a short questionnaire about the perceived efficacy of and satisfaction with the intervention program, whether they would suggest any changes to the program, and if they would recommend it to other cancer survivors. Focus groups with a subset of participations may be held to provide some qualitative information as a supplement to the questionnaire data. Together with the non-participant analysis, we will use these data to further optimize the intervention.

##### Cost effectiveness analysis

A cost-effectiveness analysis (CEA) will be performed comparing internet-based PA programs versus usual care, from a societal perspective for both breast cancer and prostate cancer. Two Markov decision models will therefore be constructed with mutually exclusive health states. The model will simulate the course of events in a breast and prostate cancer cohort, based on the PABLO data for comparing the two intervention strategies and compared to usual care. The models will first evaluate the cost-effectiveness using the time horizon of the study (12 months), and subsequently will simulate the expected costs and outcome with a lifetime horizon.

The cost-effectiveness of the internet-based PA programs versus usual care will be expressed as: [1] the effectiveness part of the equation will be based on EQ5D scores after 6 and 12 month follow-up. [2] the cost part of the equation will be expressed in the following items:

Direct costs: The intervention costs will be calculated, including health professional labour (including time spent on treatment per patient by social workers by means of the Activity Based Costing (ABC) method) [50], staff training, administration, and material costs (e.g. related to information technology). Costs of personnel involved in the interventions will be calculated by the time they are involved times the standard costs for (para)medical personnel. By means of the iMCQ, all patients will be asked to report on T1 and T2 about their use of health care services (e.g., GP, medical specialist, paramedical care etc.), prescribed medication use. Direct non-medical costs will include travel costs.

Indirect costs: This is the period over which the production loss is calculated, i.e. the time that an employer needs to replace a sick employee, will be measured by the Friction cost method. Therefore, an abbreviated version of the iMTA’s productivity costs questionnaire (iPCQ (see www.imta.nl/questionnaires)) will be administered at the same time points as the panel of questionnaires. The calculation of the average labour costs per working day will be based on the weighted average labour costs of full-time and part-time employed persons in the Netherlands. The friction costs are assumed to be 80% of wage costs [51]. Where appropriate, Dutch guidelines for costing studies will be used in applying tariffs to units of resource use [52]. As real-world implementation can differ from the research setting, various scenarios of implementation and compliance will be used for modelling, based on the method described by Retèl et al. [53].

### Data management

#### Handling and storage of data and documents

Data will be handled confidentially. A subject identification code list will be used to link the data to the subject. The key to the code will be safeguarded by the investigators (HvdW and WG) and will be saved on a computer in the work office at the AVL. The data will be coded using numbers in order enrolment, 1 / 2 / 3 etc., and the recruiting hospital will be added as a fixed code after that A / B / C. Only the investigators HvdW, WG, WvH and MS will have access to the source data. Data will be stored for at least 15 years in an office at the AVL and on a network folder especially made for research data storage.

#### Amendments

Amendments are changes made to the research after a favourable opinion by the accredited Institutional Review Board (IRB) has been given. Substantial amendments will be notified to the IRB that gave a favourable opinion. Non-substantial amendments will not be notified to the accredited IRB and the competent authority, but will be recorded and filed by the sponsor.

### Safety report

#### Temporary halt for reasons of subject safety

In accordance to section 10, subsection 4, of the Dutch law of scientific research involving human subject (WMO), the sponsor will suspend the study if there is sufficient ground that continuation of the study will jeopardise subject health or safety. The sponsor will notify the accredited IRB without undue delay of a temporary halt including the reason for such an action. The study will be suspended pending a further positive decision by the accredited IRB. The investigator will take care that all subjects are kept informed.

##### (Serious) adverse events

- Adverse events (AEs):

Adverse events are defined as any undesirable experience occurring to a subject during the study, directly related to the experimental interventions. All adverse events reported spontaneously by the subject or observed by the investigator or his staff will be recorded.

- Serious adverse events (SAEs):

A serious adverse event is any untoward medical occurrence or effect that

- results in death;
- is life threatening (at the time of the event);
- requires hospitalisation or prolongation of existing inpatients’ hospitalisation;
- results in persistent or significant disability or incapacity;
- is a congenital anomaly or birth defect; or
- any other important medical event that did not result in any of the outcomes listed above due to medical or surgical intervention but could have been based upon appropriate judgement by the investigator.

An elective hospital admission will not be considered as a serious adverse event.

SAEs that result in death or are life threatening will be reported expedited through the webportal *Toetsingonline* to the accredited IRB that approved the protocol. The expedited reporting will occur not later than 7 days after the responsible investigator has first knowledge of the adverse reaction. This is for a preliminary report with another 8 days for completion of the report. The sponsor will report the remaining SAEs twice a year in line listings.

##### Follow-up of adverse events

Exercise-related AEs will be followed until they have abated, or until a stable situation has been reached. Depending on the event, follow up may require additional tests or medical procedures as indicated, and/or referral to the general physician or a medical specialist. SAEs need to be reported till end of study within the Netherlands, as defined in the protocol.

### Statistical analysis

#### Primary study parameter

Change in weekly time spent in MVPA, as measured with the accelerometer, between baseline and 6 months will be used as the main outcome parameter. We will evaluate differences over time between intervention (blended care and web-only) and usual care with a mixed-effects linear regression model adjusted for MVPA baseline scores and stratification factors.

#### Secondary study parameter(s)

We will evaluate between-group differences over time in self-reported level of PA, fatigue, mood, and HRQOL using mixed effects linear regression models. Scores for the IPAQ, MFI, POMS, SF-36 will be calculated according to existing algorithms. Differences in mean change scores over time between the combined intervention group and the control group (blended care and web-only) will be accompanied by effect sizes. Effect sizes, defined as standardized measures of the strength of the difference in improvement between groups over time, will be calculated by subtracting the mean change scores of the control group from those of the intervention group, and subsequently dividing this by the pooled standard deviation. Effect sizes of 0.2 are considered small, 0.5 moderate, and 0.8 large [54]. The *p*-value for overall model effects will be set at 0.05, and for specific contrasts to 0.01, lowering the risk of Type I errors due to multiple testing [55]. All analyses will be conducted on an intention-to-treat basis. In addition, per-protocol analyses will be performed on patients according to compliance levels.

#### Other study parameters

##### Socio-demographic and clinical data

Descriptive statistics will be generated to describe and compare intervention and control groups, in terms of socio-demographic and clinical characteristics.

##### Moderators and mediators of PA participation

We will employ mediation analysis to assess the underlying working mechanism of the intervention with regard to the primary outcome, hypothesizing that the intervention will result in a more positive attitude towards PA, larger exercise-self efficacy, and less symptoms. This analysis will be of an exploratory nature. Also, we will explore possible moderation of the treatment effect for primary tumour type (breast or prostate) and age by adding interaction terms. To investigate which factors at T0 (pre-intervention) and T1 (post-intervention) best predict adherence to public health guidelines (at least 150 min/week of moderate to vigorous PA) at 12 months (T2), we will employ multiple regression analysis. Predictors of interest include, demographic and clinical characteristics (age, tumour type, current anti-hormonal treatment, presence of any comorbidity), stage of change at 6 months, exercise self-efficacy and behavioural beliefs [56].

##### Non-participation analysis

The baseline measures of the participants of the study will be compared to those of non-participants using chi-squared statistic for categorical variables and analysis of variance for continuous variables.

##### Compliance

Compliance will be analysed by descriptive statistics. Additional analyses will be carried out in which data relating to compliance with the program elements is taken into account. To this end, we will determine whether the level of compliance (based number of log-ins and self-reported use data) is significantly associated with the changes over time in primary and secondary outcome measures.

##### Patients’ evaluation of the intervention program

Descriptive statistics will be generated to describe the intervention and control groups in terms of patients’ evaluation of the intervention program. Outcomes will be compared using analysis of variance.

##### Cost effectiveness

The outcome of the cost-effectiveness analysis is the incremental cost-effectiveness ratio, expressed in cost / QALY, represents the additional costs required for the particular intervention to generate one additional QALY in comparison to doing nothing. These are calculated as follows:

ICER = (Costs of the intervention group – costs of the UC group) /

(QALYs of the intervention group – QALYs of the UC group)

Secondly, the ICER will be expressed in cost/MET minutes MVPA/week, representing the additional costs required for the gain in PA level/week.

State of the art health economic methods will be applied. These include the estimation of the degree of uncertainty about each input parameter and the use of probabilistic sensitivity analyses. Parameter values will be drawn randomly from the assigned distributions, using Monte Carlo simulation with 10,000 iterations. The degree of uncertainty will be illustrated by using confidence intervals for costs and health effects. Scatter-plots, confidence ellipses on cost-effectiveness planes and cost-effectiveness acceptability curves will be presented [57]. As an indication of whether an intervention will be considered cost-effective the ICER is compared to a range of ceiling ratios varying from €20 k / QALY for prevention to €80 k / QALY for severe diseases, in the Netherlands. If necessary, Value of Information (VOI) analysis will be performed to support decision-making regarding adoption and further research [58].

## Discussion

Meeting PA guidelines is important for the wellbeing of cancer survivors and is associated with considerably lower health care spending. The aim of this trial is to evaluate the (cost)effectiveness of the IPAS intervention alone or combined with physiotherapy counselling on PA levels of breast and prostate cancer survivors compared to usual care. This intervention could have important implications for the health and well-being of a large number of cancer survivors, as prostate and breast cancer are the most frequent occurring types of cancer.

This study has limitations that need to be acknowledged. The first is related to the interventions, neither of which is supervised in-person. A recent meta-analysis of individual patient data of 34 exercise RCTs has shown that such supervised interventions are more effective than unsupervised interventions in terms of quality of life and physical functioning [59]. However, since long-term maintenance of PA is necessary to optimally benefit from physical activity, providing supervised interventions is financially and practically undesirable. In the IPAS-TS intervention we offer a partly supervised intervention which could be characterized as guided self-management. The second limitation is that we will not reach every single cancer survivor, especially those that are most in need. Chances are that -given the nature of the intervention- we will not be able to recruit older, less educated, less motivated or less tech-savvy patients, whereas many of these patients may benefit most from improving their PA level. We anticipate that our non-participant analysis will help us clearly define the characteristics of these patients and will provide leads to better target these patients in future exercise programs. A third limitation may be that the internet-based intervention is prone to suffer from relatively high attrition rates, which is a common observation with internet-based behavioral interventions [60]. We anticipate that, by adding remote supervision, the compliance and the efficacy of the intervention will increase substantially. A further limitation may be that we did not chose for a forced 50–50% inclusion rate of breast and prostate cancer survivors. Since we do not expect significant differences in our outcome measures within breast and prostate cancer survivors, we use stratification on tumor type to level out possible differences. Although the degree of (in) activity is a selection criteria, it is known in behavourial studies that wide attention for healthy behaviour, can also influence the PA levels of control group patients. This “moving target” effect is difficult to anticipate on in methodological terms, but should be taken into account from the onset.

The proposed study also has several strengths, including: [1] using the IPAS intervention which was already piloted, [2] the large sample size, [3] the multicenter RCT design, [4] the intention to treat approach, [4] a relatively long term follow-up, [5] using objectively measured PA as outcome, [6] the addition of a cost-effectiveness analysis, [7] a head to head comparison of an online-only versus a partly remotely supervised program 8] a detailed analysis of the background characteristics of those who decline participation in the trial [9] targeting breast and prostate cancer survivors that have a high prevalence, [10] detailed analyses of psychological factors that may predict long-term physical activity behaviour.

In summary, we here presented the rationale and design of the PABLO trial which aims to improve PA levels of breast and prostate cancer survivors via internet-based or a minimally supervised intervention. The trial adds to the literature in several important ways (eg. cost effectiveness analysis, non-participant analysis). If proven to be (cost)effective, these interventions may be added to the standard care of breast and prostate cancer survivors to enhance their health and quality of life in a modern and efficient way.

## Additional files

Additional file 1: Overview stages of behavioural change. This figure shows an overview of stages of behavioural change. (DOCX 805 kb)

Additional file 2: Example of an advice page within the IPAS program. This image shows a page of the IPAS program with physical activity advice. (DOCX 3104 kb)

Additional file 3: Protocols for intake and telephone consults for physiotherapists. This tables shows protocols for the intake and telefone consults for the physiotherapists. (DOCX 18 kb)

## Abbreviations

(S)AE: (Serious) Adverse Event
ABC: Activity Based Costing
CEA: Cost-Effectiveness Analysis
GPS: Global Positioning System
HRQOL: Health Related Quality of Life
ICER: Incremental Cost Effectiveness Rate
iMCQ: Medical Consumption Questionnaire
IPAQ: International PA Questionnaire
IPAS: Internet Based Physical Activity Support
IPAS+TS: Internet Based Physical Activity Support + Telephone Support
iPCQ: Productivity Cost Questionnaire
IRB: Institutional Review Board
MET: Metabolic Equivalent Time
MFI: Multidimensional Fatigue Inventory
MVPA: Moderate to Vigorous Physical Activity
NKI: The Netherlands Cancer Institute
PA: Physical Activity
POMS: Profile Of Mood States
QALY: Quality Adjusted Life Year
RCT: Randomised Controlled Trial
SAE: Serious Adverse Event
SCT: Social Cognitive theory
SF-36: Medical Outcomes Study Short Form-36
TPB: Theory of Planned Behaviour
TTM: Transtheoretical Model
UC: Usual Care
WMO: Wet Medisch- Wetenschappelijk onderzoek Dutch law of scientific research involving human subject
