# Supplementary figures and images for: (Cost-)effectiveness of an internet-based physical activity support program (with and without physiotherapy counselling) on physical activity levels of breast and prostate cancer survivors: design of the PABLO trial
Source: BMC Cancer. 2018 Nov 6;18:1073. doi: 10.1186/s12885-018-4927-z (PMC6220515; doi:10.1186/s12885-018-4927-z)

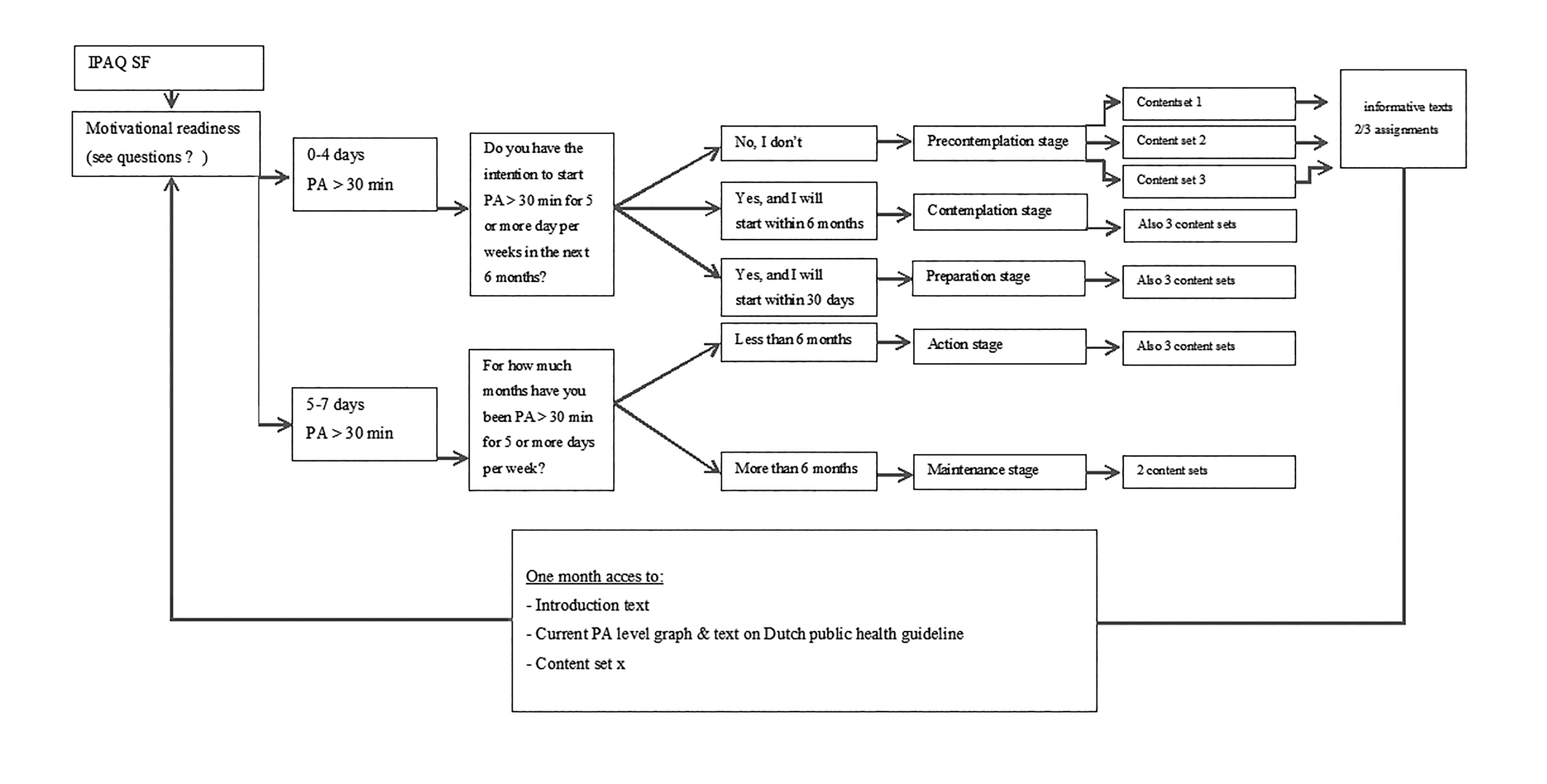
 Additional file 1 Overview stage of behavioural change

Supplement: Supplementary file 1 — Overview stages of behavioural change. This figure shows an overview of stages of behavioural change. (DOCX 805 kb) [file 12885_2018_4927_MOESM1_ESM.docx]

Additional file 2. Example of a typical IPAS page


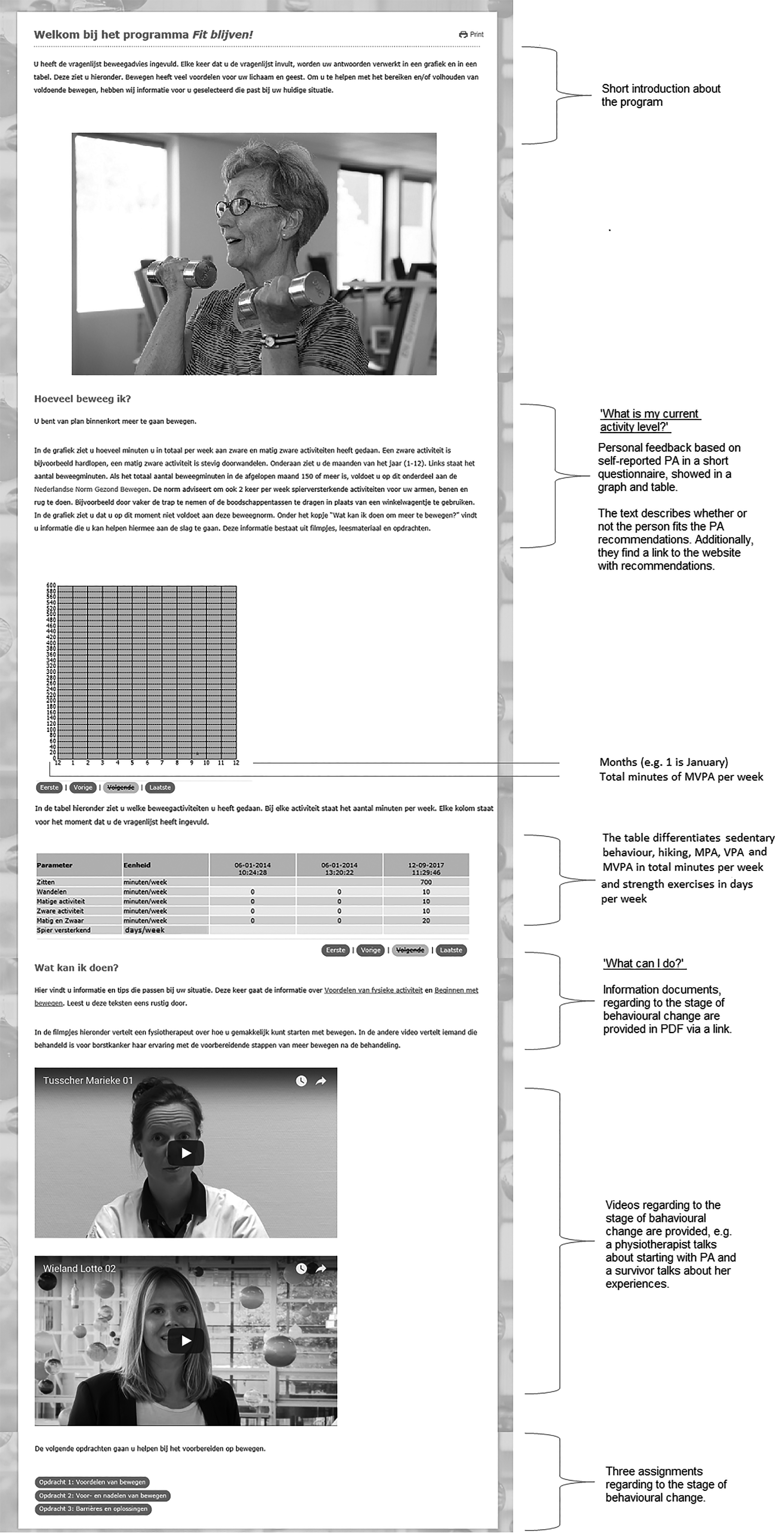

Supplement: Supplementary file 2 — Example of an advice page within the IPAS program. This image shows a page of the IPAS program with physical activity advice. (DOCX 3104 kb) [file 12885_2018_4927_MOESM2_ESM.docx]
