# Supplementary material for: (Cost-)effectiveness of an internet-based physical activity support program (with and without physiotherapy counselling) on physical activity levels of breast and prostate cancer survivors: design of the PABLO trial
Source: BMC Cancer. 2018 Nov 6;18:1073. doi: 10.1186/s12885-018-4927-z (PMC6220515; doi:10.1186/s12885-018-4927-z)
Supplement: Supplementary file 3 — Protocols for intake and telephone consults for physiotherapists. This tables shows protocols for the intake and telefone consults for the physiotherapists. (DOCX 18 kb) [file 12885_2018_4927_MOESM3_ESM.docx]

Additional file 3 Protocols for intake and telephone consults for physiotherapists.

PABLO study - Intake

Scheme patient

| Name |  |
| --- | --- |
| Date |  |

| **Elements** | **Discussed**  **(Yes/No)** | **Remarks** |
| --- | --- | --- |
| I Introduction and aim for the program |  |  |
| Discussing the intake questionnaire PACE NL |  |  |
| Movement experience moment |  |  |
| Conclusion |  | The following agreement is made:    Participant is planning to:  Preferences for call next time: |
| Particularities? |  |  |

PABLO study - Planned Call #1

Scheme patient

| Date |  |
| --- | --- |
| Enddate  Intervention | = date intake + 6,5 months |

| **Elements** | **Discussed: (Yes/No)** | **Remarks** |
| --- | --- | --- |
| Determine/appoint stage of change |  |  |
| Review on the internet-based program:  Did you read the information?  Did you do the assignments?  Do you have any questions about it?  Explanation if necessary |  |  |
| Appoint agreement last consult: |  |  |
| How did it go?   1. clarify + reflect on feelings and thoughts. 2. Empower/normalize/ explain when necessary and applicable. |  |  |
| Make new agreement with participant for next time |  |  |
| Particularities? |  |  |
